# Hope among patients discharged from an intensive care unit: A prospective cohort study

**DOI:** 10.1111/nicc.13235

**Published:** 2025-01-06

**Authors:** Mona Austenå, Tone Rustøen, Milada Cvancarova Hagen, Åse Valsø, Kjetil Sunde, Kirsti Tøien

**Affiliations:** ^1^ Department of Postoperative and Intensive Care Nursing, Division of Emergencies and Critical Care Oslo University Hospital Oslo Norway; ^2^ Department of Research and Development, Division of Emergencies and Critical Care Oslo University Hospital Oslo Norway; ^3^ Institute of Health and Society, Faculty of Medicine University of Oslo Oslo Norway; ^4^ Faculty of Health Sciences Oslo Metropolitan University Oslo Norway; ^5^ Lovisenberg Diaconal University College Oslo Norway; ^6^ Department of Anesthesiology, Division of Emergencies and Critical Care Oslo University Hospital Oslo Norway; ^7^ Institute of Clinical Medicine, Faculty of Medicine University of Oslo Oslo Norway

**Keywords:** cohort study, critical care, hope, patient, social support

## Abstract

**Background:**

Hope is important during critical illness due to the uncertainty and loss of control in the patient's life. Following intensive care, hope might provide a therapeutic effect and increase coping, leading to improved recovery.

**Aim:**

To describe the levels of hope in patients during the first year after ICU treatment, and to explore possible associations between hope and selected demographic, clinical and psychosocial factors.

**Study Design:**

This is a prospective cohort study and a predefined sub‐study of a randomized controlled trial. Adults discharged from five mixed ICUs were included. All patients were screened for post‐traumatic stress symptoms at baseline, and data on hope, post‐traumatic stress and social support were collected 3, 6 and 12 months later. Linear regression analyses and linear mixed models for repeated measurements with hope as the dependent variable were used.

**Results:**

Median age was 57 years (range 18–94), 47% were women, median length of ICU stay was 3 days (range 1–83), Simplified Acute Physiology Score II was 24 (range 0–78) and 54% received mechanical ventilation. Not having prior mental health problems (B = 1.93, 95% CI [0.90, 2.98]), lower level of post‐traumatic stress symptoms (B = −0.08, 95% CI [−0.11, −0.04]) and more social support (B = 0.37, 95% CI [0.31, 0.43]) were all independently associated with higher levels of hope during the first year after critical illness. The levels of hope were higher in the study cohort than in the general Norwegian population and remained unchanged during follow‐up.

**Conclusions:**

Patients maintained a stable level of hope throughout follow‐up. Absence of prior mental health problems, lower post‐traumatic stress symptoms and more social support after ICU discharge were associated with higher hope.

**Relevance to Clinical Practice:**

Patients' hope should be strengthened during the ICU stay through psychosocial support and care for patients with previous post‐traumatic stress symptoms and mental health problems.

**What is known about the topic**

- During intensive care treatment, hope is essential for patients to enable them to handle the insecurity and lack of control in a life‐changing situation.
- Hope is forward‐looking, and might also help patients to cope with the recovery phase after discharge from the intensive care unit.

**What this paper adds**

- Patients maintained a stable level of hope during the first year after intensive care treatment.
- Higher levels of hope throughout the follow‐up were associated with lack of previous mental health problems, lower levels of post‐traumatic stress symptoms and more social support.

## 1 INTRODUCTION

Hope is an important factor in coping with severe illness, due to the onset of sudden and unexpected insecurity and diminished control in life.^1^ Moreover, hope is universal and a relevant topic in diverse cultures,^2^ described as a multidimensional and dynamic life force comprising an expectation of achieving something good, uncertain yet possible and personally important.^3^ It is a complex construct consisting of thoughts, feelings and action, process‐oriented and might change over time,^3^ through learning and further development.^4^ Finally, hope is future‐oriented with an ability to provide strength, motivation and energy in life‐changing situations.^5^

## 2 BACKGROUND

There is limited research on hope in patients after intensive care unit (ICU) treatment. Previous empirical work has mostly focused on patients with chronic conditions, with hope as a means for enduring and coping with the future, revealing an association between higher hope levels and improved physical health.^6^ In patients with spinal injuries, the meaning of hope during the first year was to break a vicious circle, where hope gave them comfort.^7^ These conditions are unlike critically ill patients who need hope to survive present difficulties.^8^

There might be unexplored psychosocial factors that could affect hope after ICU treatment. Sense of coherence (SOC) is described as a way of coping with complex stressors in life.^9^ Patients find it difficult to cope with the rehabilitation process after critical illness, and SOC has been shown to enhance coping, mental health and quality of life,^10^ indicating that SOC could increase hope. Further, social support has been shown to preserve hope for patients during critical illness,^11^, ^12^, ^13^, ^14^ with carers' commitment and involvement of relatives in care as important factors to improve motivation and hope.^15^ However, we lack data on the impact of social support on hope after ICU treatment.

Recently, hope was found to protect against development of post‐traumatic stress disorder (PTSD) in patients with medical conditions or traumatic experiences.^16^ ICU patients might view their condition as temporary, where hope becomes a coping resource aimed at future improvement,^14^ but this has not yet been examined in patients after ICU discharge.

## 3 AIMS AND OBJECTIVES OF THE STUDY

The study aim was to describe levels of hope among ICU patients during the first year after ICU discharge, and explore possible associations between selected demographic, clinical and psychosocial factors and hope levels.

## 4 DESIGN AND METHODS

This prospective cohort study is a predefined sub‐study of a randomized controlled trial (RCT), investigating whether an intervention consisting of nurse‐led follow‐up consultations for discharged ICU patients, compared with traditional care, would reduce post‐traumatic stress symptoms (PTSS) and increase SOC.^17^ The first consultation took place in the ward, while the second and third were generally conducted by phone, all performed by specially trained ICU nurses, with content based on cognitive behavioural therapy,^18^ narrative methods and salutogenic theory. The latter builds upon a philosophy of health‐promoting factors, involving social and personal resources in addition to physical capacities.^19^

### 4.1 Setting and sample

Early after ICU discharge, we consecutively included adult (≥18 years) ICU patients treated ≥24 h at one of five medical, surgical or mixed ICUs at Oslo University Hospital (OUH), from March 2014 to December 2016. OUH is the largest hospital in Norway, offering specialized medical treatment, trauma care and organ transplantations, and its organization and treatment are comparable with hospitals in other developed countries.

**FIGURE 1:**
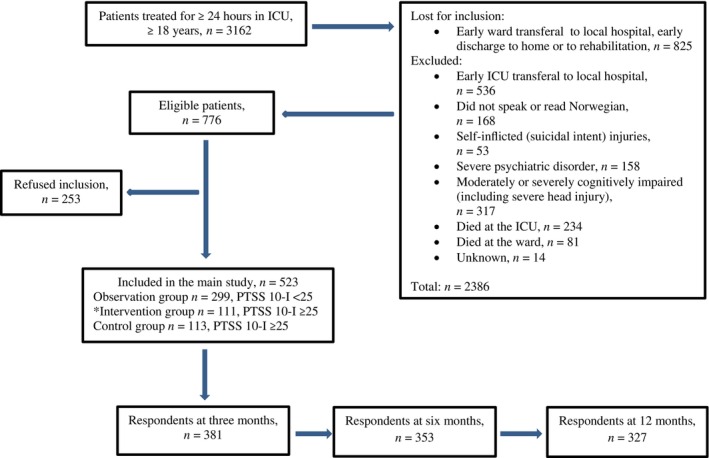
Sample at baseline and respondents in whom hope was measured at 3, 6 and 12 months after ICU discharge, from March 2014 to December 2016. *The intervention consisted of two to three nurse‐led follow‐up consultations.

A study nurse and the responsible ward nurse assessed whether discharged ICU patients could receive information and consent to participate. Patients with poor language skills, self‐inflicted injuries, severe psychiatric/psychotic disorders, terminal diseases or cognitive impairment were excluded (Figure 1).

Previously, we showed that there was no effect of the intervention regarding PTSS, SOC,^17^ pain^20^ or return to work.^21^ Importantly, the intervention was not designed to impact on hope. As there was no effect on the level of hope 3 months after ICU discharge (*B* = 0.27, 95% confidence interval (CI) [−1.28, 1.81], *p* = .732), the three groups were merged into one data set in the present study aiming to identify possible predictive factors associated with hope in the whole sample during 12‐month follow‐up.

### 4.2 Data collection tools and methods

All patients completed self‐reported paper questionnaires at the ward within a week after ICU discharge. These questionnaires were re‐sent by post 3, 6 and 12 months later with reminders, if necessary. To avoid too many questionnaires in the vulnerable time period immediately after ICU discharge, questionnaires on hope and social support were delivered to the patients 3 months after ICU discharge. All other questionnaires were collected from patients at the ward early after ICU discharge. Data were further consecutively collected at 3, 6 and 12 months for all questionnaires, including the one on hope and social support.

Hope was measured using the Herth Hope Index^22^ and Norwegian version (HHI‐N),^23^ comprising 12 items (e.g., “I believe that each day has potential”), with a scale from one (strongly disagree) to four (strongly agree) and a total score from 12 to 48 (higher score equals higher hope). This instrument has shown satisfactory validity and reliability in different patient groups,^22^, ^23^ and internal consistency measured by Cronbach's alpha^24^ was 0.86 3 months after ICU discharge. Additionally, at 3 months, hope was compared between our sample and the general Norwegian population using HHI‐N.^25^

PTSS levels were measured with the Post‐Traumatic Stress Symptoms 10‐Intensive Care Screen, part B (PTSS‐10‐B), with 10 items evaluating presence and level of PTSS during the previous 7 days, ranging from one (never) to seven (always), with a total score ranging from 10 to 70.^26^ Higher scores indicate higher stress.^26^ Patients with ≥25 points, indicating moderate to severe stress, were randomized to intervention (*n* = 111) or control (*n* = 113), and those with <25 points formed an observation group (*n* = 299). Of note, PTSS‐10‐B is not designed to diagnose PTSD.

The Sense of Coherence Scale 13 was used to measure SOC,^9^ consisting of 13 items on three dimensions: comprehensibility (making sense of adversity), manageability (resources to meet challenges) and meaningfulness (challenges are worth engagement).^17^ Each item runs from one to seven, with total scores from 13 to 91, for example, “Do you have the feeling that you don't really care what goes on around you?” (1 = very seldom or never to 7 = very often). Higher score indicates better coping ability.^19^ The scale has proven acceptability, reliability and validity in different populations, such as trauma patients.^27^, ^28^, ^29^ In the present study, Cronbach's alpha early after ICU discharge was 0.83.

Social support was measured with the revised Social Provision Scale (SPS), consisting of 16 items (e.g., “I have close relationships that provide me with a sense of emotional security and well‐being”), with responses from one (strongly disagree) to four (strongly agree) and a total score from 16 to 64. Higher score indicate more social support. The SPS contains social provisions of attachment (emotional closeness providing a sense of security), social integration (a sense of belonging to a group sharing similar interests, concerns, and recreational activities), reassurance of worth (recognition of one's competence, skills, and value by others) and opportunity for nurturance (the sense that others rely upon one for their well‐being),^30^ and has been validated in varying populations,^31^, ^32^ including ICU survivors.^33^ In our sample, Cronbach's alpha 3 months after ICU discharge was 0.82.

The following demographic characteristics were collected: educational level, current employment status (yes/no), experience of any serious life event during the previous year (deaths in family/close friends, separation/divorce, serious housing/financial problems, losing job) (yes/no) or previous mental health problems (by answering “yes/no” on five questions: troubled by symptoms without seeing a doctor, treatment by a doctor, psychologist/psychiatrist, outpatient clinic or hospital). Of note, we did not demand particular symptoms, and the five questions were merged to one variable, where “yes” to any of them was recoded as “yes”, meaning any kind of mental health problem prior to ICU admission.^18^

From the local ICU registry clinical data such as diagnosis for hospitalization, mechanical ventilation (yes/no) and length of stay in hospital and ICU (days) were collected. The American Society of Anesthesiologists Physical Status score (ASA) grades 1–4 (grade 1: Normal, healthy patient; grade 2: A patient with mild systemic disease; grade 3: A patient with severe systemic disease; grade 4: A patient with severe systemic disease that is a constant threat to life),^34^, ^35^ based on the medical record was used to measure physical health status and comorbidities prior to hospitalization. To measure illness severity, the new Simplified Acute Physiology Score (SAPS II) (range 0–163) was used, where a higher score indicates higher risk of death^36^ (Table 1). Participants' consumption of relevant medication prior to hospitalization (hypnotics, anxiolytics/antidepressants and analgesics) and medication during ICU treatment (sedatives, analgesics and antipsychotics) were also obtained.

**TABLE 1:** Demographic, clinical and psychosocial characteristics at baseline and 3 months after ICU treatment (*n* = 523).

	*n*	%
Gender		
Female	244	47
Male	279	53
Educational level		
Primary school	64	13
Secondary school	200	39
College/university	248	48
Diagnosis for hospitalization		
Trauma	93	18
Acute surgery	103	20
Elective surgery	118	23
Organ transplant	54	10
Cancer	97	19
Internal medicine	58	11
Current employment (yes)	241	47
Mechanical ventilation (yes)	280	54
Previous serious life events (yes)	173	33
Mental health problems pre‐admission (yes)	102	20

	Median	Range
Age (year)	57	18–94
American Society of Anesthesiologists Physical Status Classification (group I‐VI)	2	1–4
Length of stay hospital (days)	20	3–217
Length of stay intensive care unit (days)	3	1–83
Post‐traumatic stress symptoms 10‐intensive care screen (baseline, range 10–70)	22	10–70
Simplified acute physiology score II (range 0–163)	24	0–78

	Mean	SD
Herth hope index‐Norwegian version (3 months after ICU, range 12–48)	39	5.3
Sense of coherence (baseline, range 13–91)	69	12.4
Social provision scale (3 months after ICU, range 16–64)	56	6.5

### 4.3 Data analysis

All data were analysed using SPSS Statistics version 29 (IBM Corp, Armonk, NY, USA). Descriptive data are presented as median with range or mean with standard deviation (SD) for continuous data depending on variable distribution, and as counts and percentages for categorical variables. In the comparison with the general Norwegian population,^25^ a summary independent samples t‐test was used. The 95% CI was constructed using normal distribution approximation. Possible associations between selected variables and hope as the dependent variable were analysed using linear regression. First, univariate linear regression analyses for variables expected to be associated with level of hope 12 months after ICU discharge were performed. Thereafter, variables associated with hope with a *p*‐value ≤.1 were included in a multivariate linear regression model, which was further adjusted for gender and age. Those variables with *p*‐values <.05, in addition to gender and age, were included in a mixed‐model analysis for repeated measures, with hope assessed at 3, 6 and 12 months as the dependent variable. The results are expressed as regression estimates (B) with 95% CI. In addition, an independent t‐test to compare PTSS levels in trauma patients versus non‐trauma patients was performed. All analyses were considered exploratory, thus no correction for multiple testing was performed, and *p*‐values <0.05 were considered statistically significant.

### 4.4 Ethical and institutional approvals

The South‐Eastern Norway Regional Ethics Committee (reference number: 2012/1715) approved the study (5 July 2013), and information was provided to the data protection officer of Oslo University Hospital (reference number: 2013/9049, 12 November 2013). All patients received study information and signed a written consent before inclusion, and they could withdraw without a reason.^17^

### 4.5 Clinical trial registration

NCT02077244.

## 5 RESULTS

The median age of the 523 included patients was 57 years (range 18–94), with 47% females (Table 1). Median SAPS II score was 24 (range 0–78), 54% received mechanical ventilation and median length of ICU stay was 3 days (range 1–83) (Table 1).

Hope was measured in *n* = 381 (73%), *n* = 353 (68%) and *n* = 327 (63%) respondents at 3, 6 and 12 months, respectively (Figure 1) (*n* = 142, 170 and 196 non‐respondents, respectively). Adjusted for independent variables, the estimated mean values for hope were unchanged during the follow‐up period as quantified with *p*‐values for changes in hope over time, as presented in Table 3. The estimated mean scores for hope were 37.9 (95% CI [37.40–38.46]), 37.9 (95% CI [37.39–38.49]) and 37.7 (95% CI [37.12–38.28]) at 3, 6 and 12 months, respectively.

From the Norwegian population (4.49 million in 2000), 4000 adults were randomly drawn from the National Register, with 1912 respondents (49%). Mean hope in this population was lower^25^ than in the ICU patients at 3 months (mean 36.7, 95% CI [36.5–36.9] vs. 39, 95% CI [38.4–39.6]).

The multivariate linear regression analysis revealed that absence of mental health problems prior to ICU admission, lower level of PTSS immediately after ICU discharge and more social support at 3 months were all independent factors significantly associated with higher hope at 12 months (Table 2).

**TABLE 2:** Univariate and multivariate linear regression analyses of hope 12 months after ICU discharge.

	Univariate (*n* = 282–320^a^)			Multivariate (*n* = 268^a^)		
	*B*	95% CI	*p*‐value	*B*	95% CI	*p*‐value
Age (year)	−0.01	−0.04, 0.03	.643	−0.03	−0.07, 0.02	.233
Gender						
Male (ref.)						
Female	−0.39	−1.54, 0.75	.503	0.05	−1.05, 1.15	.926
Educational level						
Primary school (ref.)						
Secondary school	−0.07	−1.92, 1.79	.943			
College/university	1.16	−0.62, 2.94	.200			
Diagnosis for hospitalization						
Trauma (ref.)						
Acute surgery	−0.90	−2.76, 0.96	.342	0.03	−1.76, 1.83	.970
Elective surgery	−0.54	−2.30, 1.23	.551	−0.13	−1.79, 1.54	.882
Organ transplant	−0.10	−2.29, 2.09	.928	1.01	−1.17, 3.18	.364
Cancer	0.09	−1.82, 2.01	.924	0.87	−0.97, 2.70	.354
Internal medicine	2.07	−0.11, 4.24	.062	1.85	−0.42, 4.12	.110
Employment status prior hospitalization						
(ref. = not employed)	1.03	−0.12, 2.17	.079	0.12	−1.11, 1.35	.847
Mechanical ventilation						
(ref. = not mechanical ventilation)	0.08	−1.07, 1.23	.888			
Previous serious life events						
(ref. = not serious life events)	−0.78	−2.03, 0.47	.220			
Mental health problems pre‐admission						
(ref. = not mental health problems)	−4.98	−6.45, −3.52	<.001	−2.53	−4.26, −0.81	.004
ASA‐PS (group I‐VI)	−0.20	−0.88, 0.48	.558			
LOS hospital (days)	0.00	−0.02, 0.03	.866			
LOS ICU (days)	0.05	−0.01, 0.10	.116			
PTSS 10‐I (baseline, range 10–70)	−0.16	−0.20, −0.12	<.001	−0.08	−0.13, −0.02	.006
SAPS II (range 0–163)	0.04	−0.01, 0.09	.088	0.02	−0.03, 0.07	.487
SOC (baseline, range 13–91)	0.17	0.12, 0.22	<.001	0.05	−0.02, 0.11	.161
SPS (3 months after ICU, range 16–64)	0.40	0.32, 0.48	<.001	0.28	0.19, 0.38	<.001
Medication in ICU						
(ref. = no medication)						
Benzodiazepines (yes/no)	−0.58	−1.73,0.57	.318			
Opioids (yes/no)	−0.09	−2.26,2.08	.935			
Antipsychotics (yes/no)	−0.60	−2.51,1.30	.534			
Dexmedetomedine (yes/no)	−0.17	−1.62,1.27	.815			
Clonidine (yes/no)	0.37	−2.06,2.79	.767			
Regional analgesics (yes/no)	−1.03	−2.18,0.11	.076	0.45	−0.79, 1.70	.472
Medication before admission hospital						
(ref. = no medication)						
Hypnotics (yes/no)	−2.45	−3.72, −1.18	<.001	−0.31	−1.72, 1.10	.667
Anxiolytics/antidepressants (yes/no)	−2.45	−3.91, −0.99	.001	0.10	−1.58, 1.78	.908
Analgesics without prescription (yes/no)	−0.66	−1.89, 0.57	.291			
Analgesics with prescription (yes/no)	−0.50	−1.74, 0.74	.425			

Abbreviations: ASA‐PS, American Society of Anesthesiologists Physical Status Classification; LOS, Length Of Stay; ICU, Intensive Care Unit; PTSS, Post‐Traumatic Stress Symptom 10‐Intensive Care Screen; SAPS, Simplified Acute Physiology Score, SOC, Sense of coherence, SPS, Social Provision Scale.

^a^ Less than the 381 respondents at three months due to non‐respondents and missing of some variables.

The mixed‐model analysis for repeated measurements (*n* = 374) revealed a significant association between the same three independent variables and hope at 12‐month follow‐up (Table 3). Lack of previous mental health problems increased hope by two points (*B* = 1.93, 95% CI [0.90, 2.98]). For every 10‐unit decrease in the PTSS scale early after ICU discharge, hope increased by about one point (B = −0.08, 95% CI [−0.11, −0.04]). Hope level increased by four points for every 10‐unit increase on the social support scale assessed at 3 months (*B* = 0.37, 95% CI [0.31, 0.43]) (Table 3).

**TABLE 3:** Mixed‐model analysis for repeated measurements, with hope at 3, 6 and 12 months after ICU treatment as dependent variable (*n* = 374).^a^

Age (year)	*B*	95% CI	*p*‐value
	−0.02	−0.04, 0.00	0.089
Gender			
Male (ref.)			
Female	−0.13	−0.87, 0.62	0.738
Mental health problems pre‐admission			
No (ref.)			
Yes	−1.93	−2.98, −0.90	<0.001
PTSS 10‐I (baseline, range 10–70)	−0.08	−0.11, −0.04	<0.001
SPS (three months after ICU, range 16–64)	0.37	0.31, 0.43	<0.001
Hope (HHI‐N, range 12–48) over time			
3 months			
6 months	0.23	−0.24, 0.70	0.341
12 months (ref.)	0.24	−0.14, 0.63	0.214

*Note*: Demographic and psychosocial characteristics after ICU discharge (baseline) and 3 months later.

Abbreviations: HHI‐N, Herth Hope Index‐Norwegian version; PTSS, post‐traumatic stress symptom 10‐Intensive Care Screen; SPS, Social Provision Scale.

^a^ Less than the 381 respondents at 3 months due to non‐respondents and missing of some variables.

There was no difference in PTSS level between trauma patients (mean 24.25) and non‐trauma patients (mean 25.88) difference (*d*) = −1.63, *p* = .488.

## 6 DISCUSSION

The present study is the first to investigate the trajectory of hope during the first year after ICU treatment. We found a stable level of hope among discharged ICU patients during this period. Higher levels of hope were associated with absence of mental health problems prior to ICU admission, lower level of PTSS immediately after ICU discharge and more social support 3 months later. The levels of hope 3 months after ICU discharge were higher than in the general population.^25^

That hope among ICU survivors was higher 3 months after ICU discharge than in the general Norwegian population is interesting and might indicate that having survived critical illness strengthens hope. This is supported by a recent study exploring hope after critical illness, where patients interviewed within 4 days of ICU discharge felt an impetus and a desire to improve the further direction of their lives.^37^ Moreover, our patients had stable levels of hope throughout the first year after ICU discharge. This concurs with the findings of how hope during critical illness helps patients make sense in life‐changing situations with high uncertainty and low control.^1^ The presence of hope throughout the ICU stay has also previously been described as future‐oriented towards improvement, with the aim to get life back to normal.^8^, ^11^ Just as hope helps patients to cope with chronic diseases and treatment, leading to improved health,^6^ strengthened hope might seem to persist even after ICU treatment. Enhanced hope was also seen in patients interviewed already two to 4 days after ICU discharge, where hope was inspired by mental coping, a belief that difficulties led to growth, a desire to live and a wish for a positive outcome.^12^

The association between prior mental health problems and level of hope during the first year after ICU discharge concurs with results from the general population, reporting lower levels of hope for those with psychiatric disorders than patients with other chronic diseases.^25^ As much as 20% of our patients had mental health problems prior to ICU admission, which is higher than the 6.4% in the Norwegian general population.^25^ Notably, college students with high rates of depression, anxiety and distress reported that depression and negative life events were less intense for those who were more hopeful.^38^ Previous research on ICU patients with mental health problems prior to ICU admission has mostly focused on higher risk of organ failure, mortality^39^ and development of delirium,^40^ and not on the association between mental health and hope. We can only speculate if those with previous mental problems have reduced willingness and strengths to fight in the rehabilitation period, which might further affect their capacity and reducing their immune system being prone to infections and more complications.

We also found that lower PTSS levels early after ICU discharge were associated with increased levels of hope during the follow‐up period. In a recent meta‐analysis, Gallagher et al.^16^ described a moderate relationship between hope and a lower level of PTSD in 20 cross‐sectional studies among different patient groups, but only two prospective studies reported a small to moderate longitudinal effect of hope on PTSD. In comparison, questioning the clinical relevance of the results from the present study, the effect of PTSS on hope might be limited as hope only increased by one point when PTSS decreased by 10 points. Noteworthy, the present data did not indicate that trauma patients had higher levels of PTSS than non‐trauma patients.

The association between higher level of hope during the first year after ICU discharge and more social support was also interesting. Previous research on nurses' perspectives and strategies to inspire and promote hope in patients and their families throughout critical illness emphasizes the importance of maintaining hope with external help.^11^, ^12^, ^13^, ^14^, ^15^ De Silva et al.^15^ reported from qualitative interviews with nurses the importance to offer personalized information and support for ICU patients and their families to increase hope, but that patients might lose hope if the information is inadequate or too comprehensive. Nurses must encourage family members to visit ICU patients and ensure that they provide important support, also in terms of motivating and encouraging hope. The family knows the patient's background, and should therefore be actively encouraged to reach out to weak patients unable to receive or handle information about their condition.^15^ A previous study also shown that health care personnel encouraging even small physical improvements in patients and acknowledging relatives' contribution in interaction with patients are significant factors in maintaining hope.^37^ Similarly, Rustoen et al.^41^ found that patients with breast cancer had increased level of hope after receiving an intervention to increase hope. Obviously, there is a need for more studies on the long‐term impact of social support on levels of hope after ICU discharge, and RCTs are warranted.

We did indeed find an association in the univariate regression analysis between SOC assessed immediately after discharge from the ICU and hope 12 months later, but not when adjusted for other independent variables and not throughout the follow‐up period. In contrast to our findings, Lin et al.^42^ found that SOC was positively associated with the level of hope over time among older patients in a cross‐lagged study. In another observational, cross‐sectional, multi‐centre study, stronger family SOC was independently associated with hope, both among patients living with palliative cancer and in their family members.^43^ The present different results might be due to the fact that these two concepts have their own unique characteristics, and although they have the potential to protect against stressful situations, they still have separate roles in dealing with such events.^44^

## 7 LIMITATIONS

The present study has several limitations. We were not permitted to retrieve data on patients refusing participation or transferred to other hospitals prior to inclusion. Thus, our sample might not be representative for all discharged ICU patients. In addition, this is a single‐centre study in a single jurisdiction, which reduces generalizability. Similar studies should be conducted in other parts of the world.

Several patients were too weak early after ICU discharge and were lost for inclusion. The level of hope among these patients and those dying (*n* = 64, 12%) during follow‐up was not compared with hope in the survivors (*n* = 459, 88%). How answers from these patients could have affected the results is unknown, but an overall poorer condition among those not included or those who died during follow‐up might have reduced the mean level of hope.

Further, the use of self‐report questionnaires has their weakness by not providing a complete and accurate picture of the patient's situation,^45^ and this could have been improved using direct patient interviews.^46^

Patients with severe psychiatric/psychotic disorders at inclusion were excluded. As previous mental health problems have been shown to affect hope, this could have biased the results. However, only patients with severe mental disorders were excluded, and still 20% of the patients reported mental health problems, justifying generalizability.

In addition, earlier measurements on hope than 3 months after ICU discharge might have indicated different levels of hope. Even if our outcome was on hope and social support an independent factor, the association might as well go the opposite way. However, using statistical methodology, we could only identify associations and no causal relationships. The revised SPS^30^ does not give any information about the patients' family members, or from whom they received social support, only a total score for perceived support. We do not know whether lower social support might be associated with living separated from one's close family members.

HHI‐N is designed for measuring hope in clinical settings^23^ in different patient groups, but as far as we know, it has not previously been used in ICU patients. The estimated effect of PTSS on hope during the first year after ICU discharge was significant, but the point estimate was low, questioning its clinical relevance. Finally, this is the fourth sub‐study of a previous RCT,^17^ and the data collection is quite dated (until 2018). However, we do not assume that the phenomenon under investigation has changed and thus consider the study and results still as relevant.

## 8 IMPLICATIONS AND RECOMMENDATIONS FOR PRACTICE

The present study emphasizes the importance of maintaining patients' hope during the ICU stay. This could be provided by nurses with commitment, adapted information and detailed explanations, with the result that patients achieve a sense of taking back control in life and a feeling of improvement.^8^, ^11^ Offering psychosocial support is one way of strengthening hope during and after ICU treatment, and this could involve more flexible visiting hours for patients' families.^15^ Nurses seem to possess the strategies to maintain patients' hope, but how this knowledge is acquired is yet scarcely investigated. Our results indicate that during and after the ICU stay, particular attention should be directed towards patients with PTSS and prior mental health problems, including early reinstatement of regular medication for mental disorders. Finally, measuring patients' hope after ICU discharge might be a method to identify vulnerable patients with special follow‐up needs.

## 9 CONCLUSION

The levels of hope were stable and maintained for 1 year in discharged ICU patients. The factors associated with higher levels of hope were absence of prior mental health problems, lower level of PTSS early after ICU discharge, and more social support 3 months later.

## Data Availability

Research data are not shared.
